# Dissecting the molecular dance: c-di-GMP, cAMP-CRP, and VfmH collaboration in pectate lyase regulation for *Dickeya dadantii*—unveiling the soft rot pathogen’s strategy

**DOI:** 10.1128/spectrum.01537-23

**Published:** 2023-10-09

**Authors:** Biswarup Banerjee, Xiaochen Yuan, Ching-Hong Yang

**Affiliations:** 1 Department of Biological Sciences, University of Wisconsin-Milwaukee, Milwaukee, Wisconsin, USA; 2 Department of Plant Pathology, Entomology and Microbiology, Iowa State University, Ames, Iowa, USA; Northwestern University, Chicago, Illinois, USA

**Keywords:** quorum sensing, c-di-GMP, VfmH, cAMP-CRP, *Dickeya dadantii*, pectate lyase

## Abstract

*Dickeya dadantii* is a Gram-negative bacterium that causes soft rot diseases in a variety of plants. This pathogen secretes pectate lyase (Pel) enzymes to degrade the plant cell wall, a process controlled by the bacterial second messenger cyclic diguanylate monophosphate (c-di-GMP). VfmH is a Fis-family response regulator of the Vfm quorum sensing system, which has been previously reported to positively regulate *pel* transcription. Our results demonstrated that VfmH upregulated the production of Pel at low c-di-GMP levels, which was suppressed by c-di-GMP *via* deleting gene *egcpB*, encoding a phosphodiesterase that degrades c-di-GMP. Multiple sequence alignments between FleQ, a known c-di-GMP binding protein, and VfmH revealed several potential c-di-GMP binding motifs. We further validated these motifs *via* single amino acid substitutions. Indeed, derivatives of VfmH, including VfmH^R195A^, VfmH^R279A^, and VfmH^R344A^, abolished the c-di-GMP binding ability and the corresponding regulation in Pel. We found that VfmH exhibited ATPase activity, and this could be hindered by c-di-GMP binding. Interestingly, our results showed that VfmH interacted with CRP, suggesting that the cyclic AMP (cAMP) signaling pathway is also involved in the VfmH-mediated Pel regulation. Taken together, our study suggested that *D. dadantii* amalgamates both c-di-GMP and cAMP signaling networks along with the Vfm quorum sensing pathway to control Pel.

**IMPORTANCE:**

Bacteria respond to environmental changes and adapt to host systems. The response regulator VfmH of the Vfm quorum sensing system regulates a crucial virulence factor, pectate lyase (Pel), in *Dickeya dadantii*. At high c-di-GMP concentrations, VfmH binds c-di-GMP, resulting in the loss of its activation property in the Pel and virulence regulation in *D. dadantii*. VfmH binds to c-di-GMP *via* three conserved arginine residues, and mutations of these residues eliminate the c-di-GMP-related phenotypes of VfmH in Pel synthesis. Our data also show that VfmH interacts with CRP to regulate *pelD* transcription, thus integrating cyclic AMP and c-di-GMP signaling pathways to control virulence in *D. dadantii*. We propose that VfmH is an important intermediate factor incorporating quorum sensing and nucleotide signaling pathways for the collective regulation of *D. dadantii* pathogenesis.

## INTRODUCTION

*Dickeya dadantii,* a member of the *Enterobacteriaceae* family, is a bacterial plant pathogen that causes soft rot, wilt, and blight diseases in many economically important crops, such as tomatoes and potatoes (1). This pathogen infects the apoplast of plant tissue, relying on its ability to secrete cell wall degrading enzymes (CWDEs) *via* the type II secretion system (T2SS). Major CWDEs identified are pectate lyases (Pels), cellulases, proteases, and polygalacturonases (2 – 4), by which Pels play a dominant role in degrading pectin in the plant cell wall. A number of genes have been found that encode Pel in the *D. dadantii* genome, including *pelA*, *pelB*, *pelC*, *pelD*, *pelE,* and *pelZ*; PelD is considered one of the major endo-Pel enzymes that affect low methoxylated homogalacturonan and trigger the catalysis of β-elimination at random galactosidic bonds (5, 6). Besides CWDEs, several virulence factors, such as the type III secretion system, motility, and biofilm, have been identified in *D. dadantii* (1, 7). However, it remains mostly unclear how *D. dadantii* senses the environmental and host signals, thereby modulating the expression of virulence-related genes.

Bis-(3′−5′)-cyclic dimeric guanosine monophosphate (c-di-GMP) is a universal bacterial second messenger that has been reported to regulate various cellular processes such as Pel production and virulence in *D. dadantii* (8 – 10). A group of enzymes, namely diguanylate cyclases (DGCs), synthesize c-di-GMP from two GTP molecules. On the other hand, phosphodiesterases (PDEs) hydrolyze c-di-GMP into 5′-phosphoguanylyl-(3′−5′)-guanosine (pGpG), which is later converted to two molecules of guanosine monophosphates. Together, these two kinds of enzymes control the intracellular c-di-GMP levels in most bacteria. In general, c-di-GMP regulates cellular behaviors by binding to a wide variety of effectors, such as proteins containing PilZ-domain, GGDEF domain proteins with an I-site, proteins with degenerate GGDEF or EAL domain, and RNA riboswitches (11, 12). These physical interactions then enable the regulation of bacterial virulence and behaviors mediated by the c-di-GMP effectors. In *D. dadantii,* two PDEs, EcpC and EGcpB, and two DGCs, GcpA and GcpL, that control Pel production have been reported (6, 9, 10, 13), but c-di-GMP binding effectors remain largely unknown.

Quorum sensing (QS) is the bacterial cell-to-cell communication apparatus that uses small signaling molecules or peptides to modulate virulence and host colonization in a number of bacterial plant pathogens (14). In *D. dadantii,* the Vfm (Virulence Factors Modulating cluster) QS system is one of the major QS systems other than the classic Exp (Acyl Homoserine Lactone-based) QS system that has been reported to date (15). The two-component system VfmI/VfmH recognizes the *vfm* extracellular signal molecules. VfmH, a Fis-family response regulator, activates the expression of the master regulator VfmE, which, in turn, activates the transcription of *vfm* genes and CWDEs (16). The Vfm QS system regulates a number of virulence factors in the *Dickeya* genus (15 – 19). For example, VfmH has been reported to regulate virulence and motility in *D. dadantii* and *D. zeae*, the major cause of stalk rot in corn and foot rot in rice (16, 17). In this study, we showed that VfmH upregulated Pel under a low c-di-GMP condition, whereas under high c-di-GMP conditions, this activity was repressed. VfmH bound to c-di-GMP, and our data proved that three arginine residues were essential for this interaction. Further analysis demonstrated that VfmH derivative proteins that were immune to c-di-GMP failed to regulate Pel. We also found that VfmH exhibited ATPase activity, and the addition of exogenous c-di-GMP inhibited this activity in an *in vitro* assay. Lastly, through a pull-down assay, we identified CRP, the Catabolite Repressor Protein that binds cyclic AMP (cAMP), as an interacting partner of VfmH.

## RESULTS

### VfmH predicted model, domain organizations, and motifs

**Fig 1:**
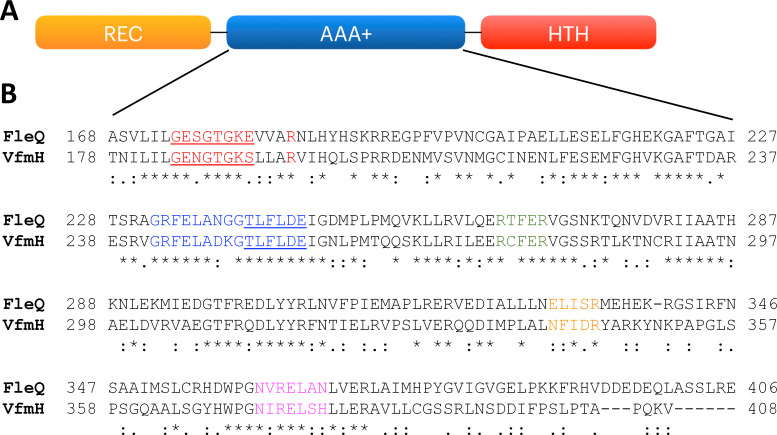
Bioinformatic analysis of VfmH. (**A**) InterPro domain search of VfmH shows the N-terminal signal receiving REC domain, AAA+ domain with potential ATPase activity and σ^54^ interaction, and a Fis-like homeobox HTH domain at the C-terminal end for potential DNA binding. (**B**) Multiple sequence alignments of AAA+ domains of VfmH revealed conserved sequences relative to FleQ from *P. aeruginosa*. Different motifs are labeled as follows: (i) Walker-A motif (red and underlined); (ii) Walker-B motif (blue and underlined); (iii) Post-Walker A arginine residue (red); (iv) RxxxR motif (green); (v) NxxxR motif (orange); and (vi and vii) potential σ^54^ interaction motifs (blue and magenta). The labels below the amino acid sequence indicate the following: “*” indicates identical amino acid, “:” indicates conserved substitution, “.” indicates semi-conserved substitution, and no label indicates non-conserved substitution.

We conducted a domain and protein family search of VfmH using InterPro (20) and found that VfmH contained three domains, including an N-terminal REC (response regulator receiver) domain, an AAA+ (ATPase) domain, and a C-terminal Fis-family like helix-turn-helix (HTH) domain (Fig. 1A). To determine the distant evolutionary relationship of the VfmH AAA+ domain, we performed a Position-Specific Iterative (PSI)-BLAST and found that the AAA+ domain of VfmH shared high similarity with the AAA+ domain of FleQ, a known c-di-GMP effector from *Pseudomonas aeruginosa* (21 – 24). Multiple sequence alignment analyses were performed between these two AAA+ domains using ClustalO, and our data showed that highly conserved motifs within the AAA+ domain, including Walker A and B motifs for the ATPase activity, σ-factor interacting motifs, and potential c-di-GMP binding regions/residues (arginine immediately after the Walker A motif, RxxxR motif, and NxxxR motif), were found (Fig. 1B). It is worth noting that a semi-conserved substitution of asparagine (N) instead of glutamic acid (E) was observed in VfmH (NxxxR) in comparison to FleQ (ExxxR) with conserved arginine residue at the end (Fig. 1B). Together, the above observations indicate that VfmH has the ability to bind nucleotides such as ATP and c-di-GMP. Furthermore, our findings suggest that VfmH may have the potential to interact with σ-factors. However, to determine whether and how these putative interactions impact the function of VfmH, further investigation is needed.

### VfmH binds c-di-GMP and three arginine residues are essential for the interaction

**Fig 2:**
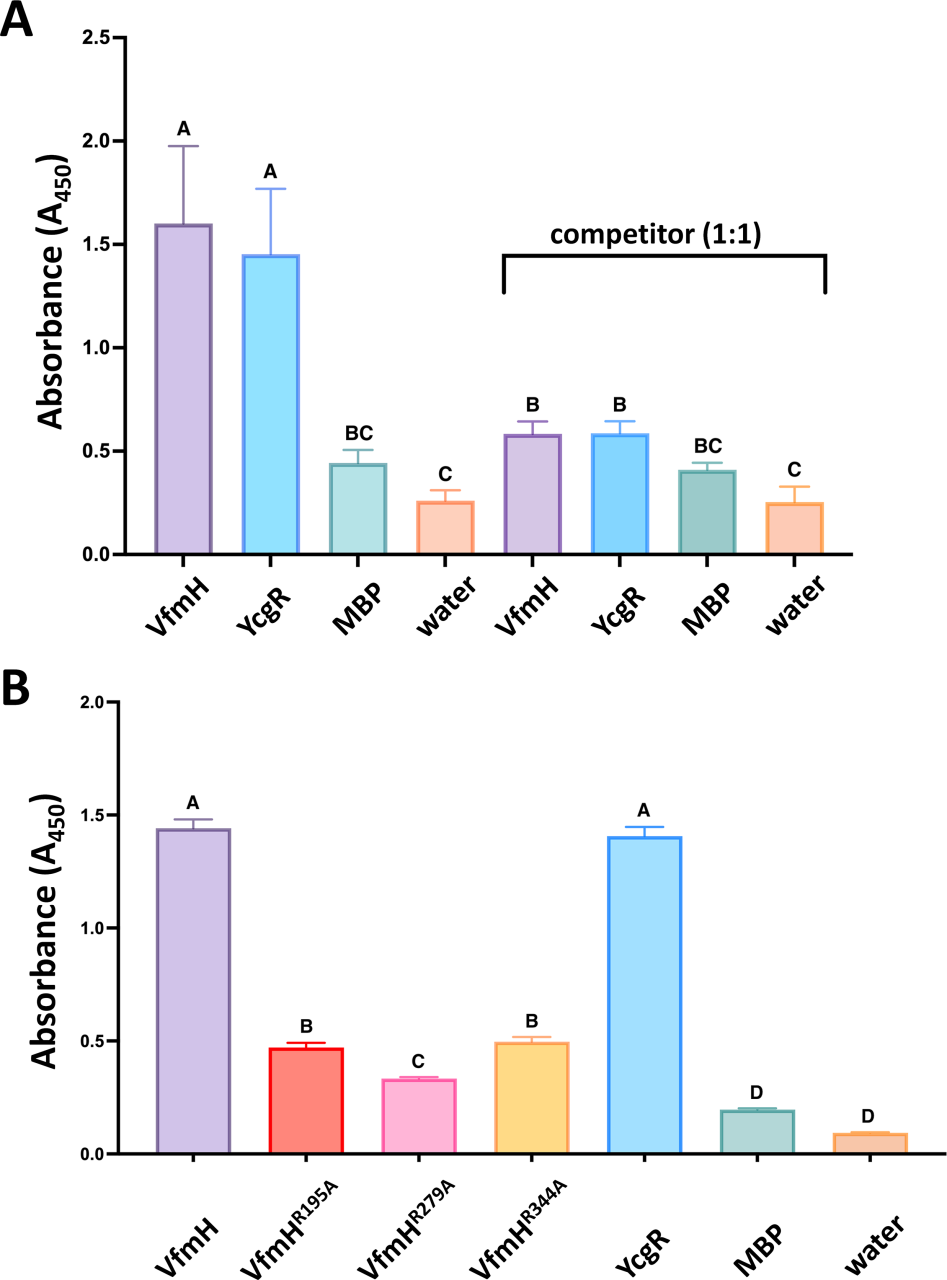
VfmH binds c-di-GMP and mutations in R195A, R279A, and R344A eliminate the interaction. The interaction of c-di-GMP and VfmH was tested by ELISA. (**A**) Absorbance at 450 nm was observed in VfmH with YcgR. MBP and sterile water were used as controls. For the competition assay, a similar experiment was conducted by adding non-biotinylated c-di-GMP in a 1:1 ratio. (**B**) Absorbance at 450 nm was observed in VfmH, VfmH^R195A^, VfmH^R279A^, and VfmH^R344A^, with YcgR. MBP and sterile water were used as controls. Values represent the mean absorbance of two independent experiments, and each experiment had triplicates. Error bars indicate the standard error of the mean. Different upper-case letters above the bars indicate statistical significance across different groups, whereas the same letters signify no statistical significance among the treatment groups (*P* < 0.05) by one-way ANOVA.

Our previous study demonstrated that VfmE is a c-di-GMP binding transcription factor that controls Pel production in *D. dadantii* (18). Here, we hypothesized that VfmH is likely also a c-di-GMP binding protein due to our finding that VfmH contains several conserved c-di-GMP binding motifs. To investigate the potential interaction between VfmH and c-di-GMP, we carried out an enzyme-linked immunosorbent assay (ELISA) using biotinylated c-di-GMP as described previously (18). Both VfmH and YcgR, a known c-di-GMP binding protein in *D. dadantii* (25), showed high levels of absorbance at 450 nm (Fig. 2A), suggesting that VfmH indeed binds c-di-GMP *in vitro*. As negative controls, the absorbance was barely detected using Maltose Binding Protein (MBP), which has been shown previously that does not bind c-di-GMP (18, 26), or water (Fig. 2A). Next, we added an equal concentration of unlabeled c-di-GMP for a competition assay to confirm our observation. A reduced absorbance in both VfmH and YcgR samples was observed, but no reduction was observed in those containing MBP or water (Fig. 2A).

Since arginine (Arg) residues are often responsible for the binding to c-di-GMP (18, 22), we determined the role of three Arg residues (namely Arg^195^, Arg^279^, and Arg^344^) from three regions, including the post-Walker A Arg residue, the second Arg residue from RxxxR motif, and the Arg residue from NxxxR motif, respectively, for their ability to bind c-di-GMP. For this purpose, we carried out a single amino acid substitution in VfmH by changing these Arg residues to alanine. The recombinant VfmH^R195A^, VfmH^R279A^, and VfmH^R344A^ proteins were tested for c-di-GMP binding. As expected, YcgR and VfmH showed high 450 nm absorbance values, but significantly reduced values were observed for VfmH^R195A^, VfmH^R279A^, and VfmH^R344A^, similar to those of MBP or water (Fig. 2B). Taken together, the above observation confirms that the Arg^195^, Arg^279^, and Arg^344^ residues in VfmH are important for c-di-GMP binding.

### The ATPase activity of VfmH is affected by c-di-GMP

**Fig 3:**
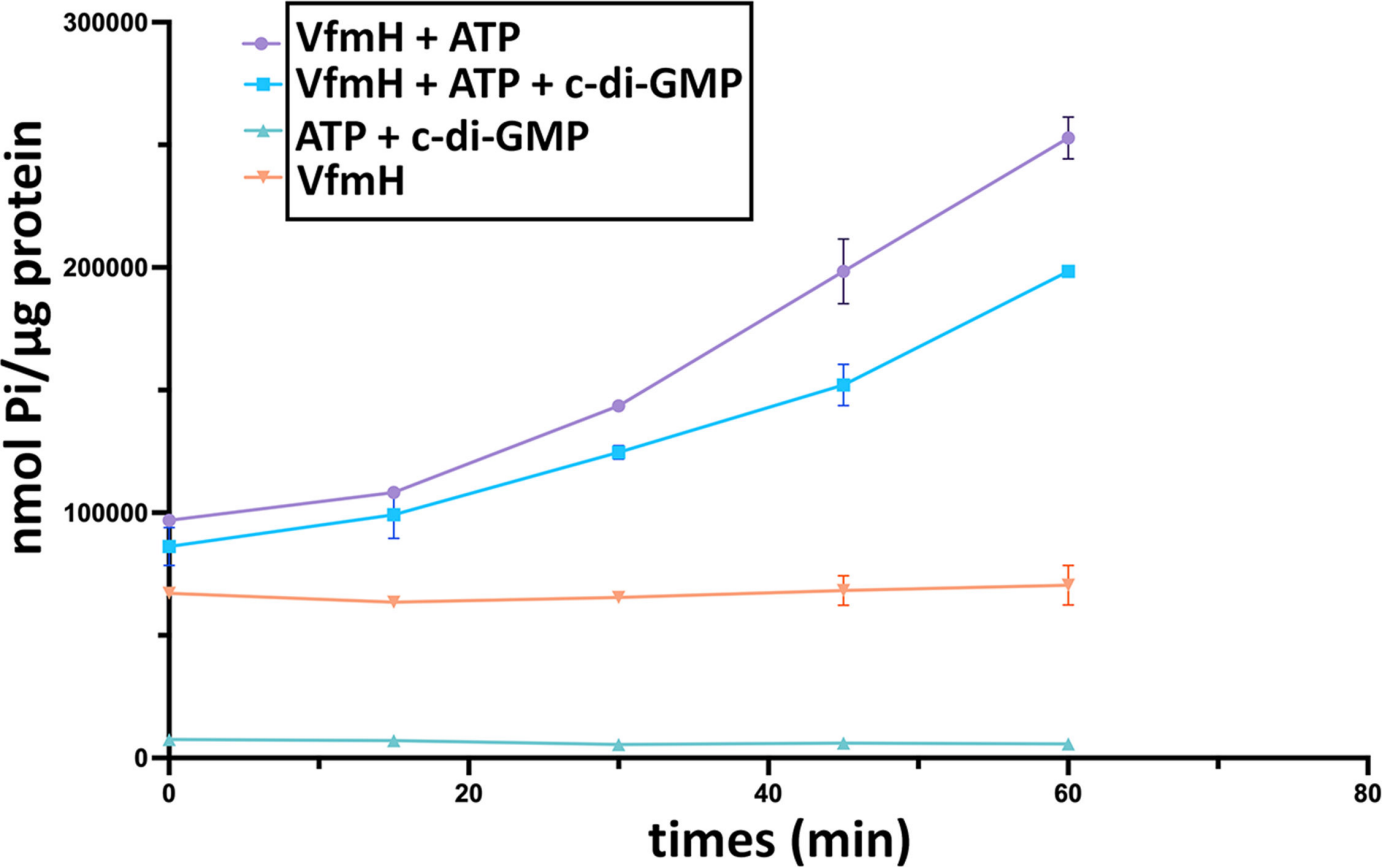
The ATPase activity of VfmH is affected by c-di-GMP. The ATPase activity of VfmH was measured using the Malachite green phosphate detection method. The ATPase activity of VfmH in the presence or absence of c-di-GMP was measured over 0, 15, 30, 45, and 60 min. VfmH protein and the nucleotides (ATP and c-di-GMP) were used as controls. Values at each time point represent the mean value of one experiment in triplicate. The values plotted in the graph represent a non-linear fit model of XY replicate data. Error bars indicate standard deviation as measured by descriptive statistics.

VfmH contains the AAA+ domain (17, 27) (Fig. 1A), in which the Walker A motif potentially binds ATP while the Walker B motif hydrolyzes it (28) (Fig. 1B). To investigate whether VfmH exhibits ATPase activity, we determined the ATP hydrolysis activity of VfmH *via* mixing 1 µM purified VfmH protein with 1 mM ATP, followed by the measurement of inorganic phosphate (Pi) produced at different time points (0, 15, 30, 45, and 60 min). VfmH without the addition of ATP was used as a negative control. We observed an increased Pi concentration over time when ATP was added to VfmH compared to the negative control, suggesting that VfmH hydrolyzes ATP (Fig. 3). Previous study with FleQ in *P. aeruginosa* suggested that c-di-GMP binding inhibits ATPase activity of the protein (22, 23). To elucidate the effect of c-di-GMP binding to VfmH on its ATPase activity, 100 µM c-di-GMP was added to the mixture of VfmH and ATP. The ATPase activity reduced gradually over time in the presence of c-di-GMP, with the calculated rate of Pi generated at 1871.16 ± 133.64 nM per 1 µg VfmH per minute relative to 2597.99 ± 111.25 nM Pi generated in samples containing only VfmH and ATP (Fig. 3), an approximately 28% of reduction. The sample containing only VfmH showed a slightly higher Pi baseline because of the phosphate salts present in the elution buffer which was used in the purification process (Fig. 3). Nevertheless, our data suggest that VfmH hydrolyses ATP, and this activity is moderately hindered by competitive inhibition from c-di-GMP.

### c-di-GMP plays a pivotal role in VfmH-mediated Pel production

**Fig 4:**
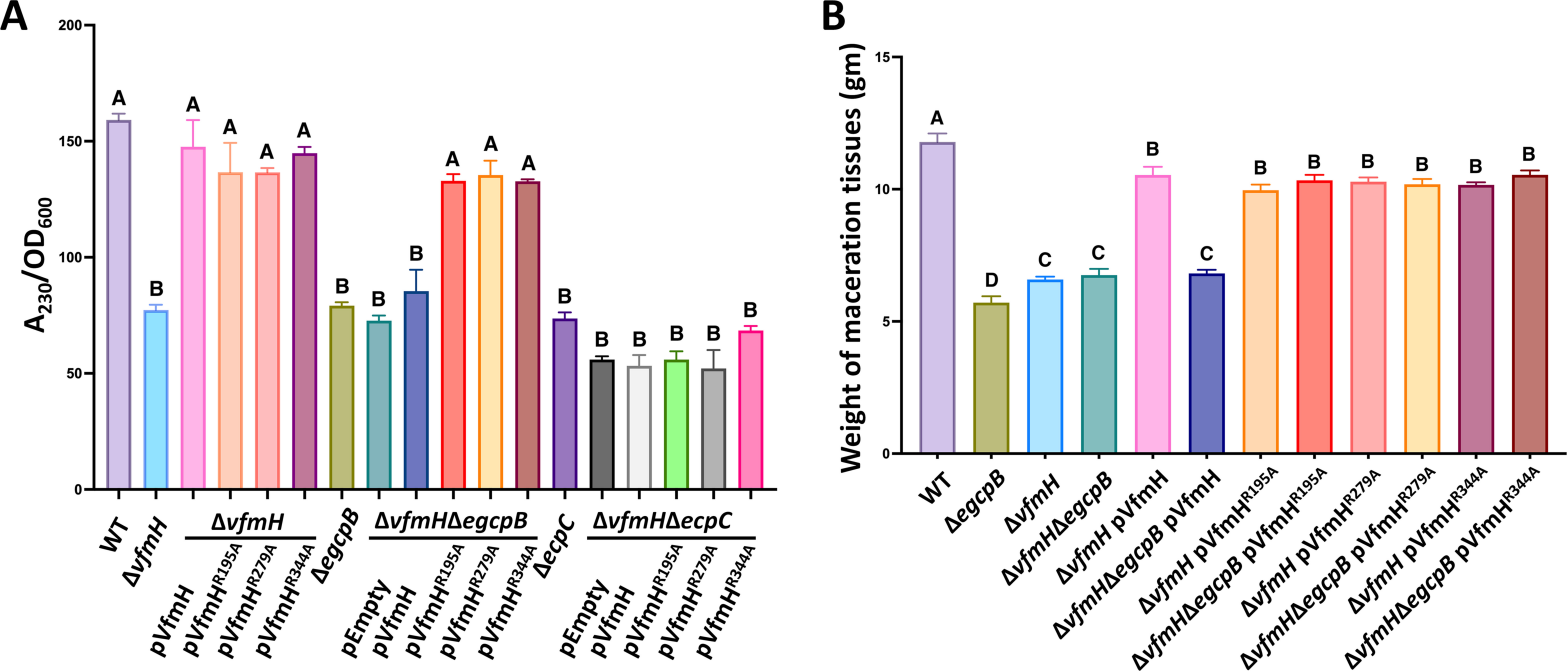
VfmH^R195A^, VfmH^R279A^, and VfmH^R344A^ recover the Pel production. (**A**) Pel activities of WT and mutants were measured. Complementation of Pel activity was done in Δ*vfmH,* Δ*egcpB,* Δ*vfmH*Δ*egcpB,* Δ*ecpC*, and Δ*vfmH*Δ*ecpC* bearing low copy number pCL1920:*vfmH,* pCL1920:*vfmH* ^R195A^, pCL1920:*vfmH* ^R279A^, or pCL1920:*vfmH* ^R344A^. Empty pCL1920 plasmid harboring strains were used as controls. The A_230_ value measuring the Pel activity was normalized by the growth of the individual strains in the broth (OD_600_). Values represent the mean Pel activity of three independent experiments, and each experiment had triplicates. Error bars indicate the standard error of the mean. Different upper-case letters above the bars indicate statistical significance across different groups, whereas the same letters signify no statistical significance among the treatment groups in LB medium for 16 h (*P* < 0.05) by one-way ANOVA. (**B**) The weights of the necrotic tissues in the potato host were measured in WT, Δ*vfmH,* Δ*egcpB,* Δ*vfmH*Δ*egcpB* mutants, and the complementation strains. Values represent the mean of three independent experiments, and each experiment had triplicates. Error bars indicate the standard error of the mean. Different upper-case letters above the bars indicate statistical significance across different groups, whereas the same letters signify no statistical significance among the treatment groups for 24 h (*P* < 0.05) by one-way ANOVA.

Two PDEs, EcpC and EGcpB, have been reported to upregulate Pel production by degrading c-di-GMP, and deletion of *ecpC* or *egcpB* significantly increases the cellular c-di-GMP levels (9, 10). VfmH was reported to positively regulate Pel production in *D. zeae* (17). We showed that VfmH could bind c-di-GMP (Fig. 2). Therefore, we hypothesized that c-di-GMP may interact with VfmH to further regulate Pel. To test this hypothesis, we constructed various single and double deletion mutants, including Δ*vfmH*, Δ*vfmH*Δ*ecpC*, and Δ*vfmH*Δ*egcpB*, and tested their extracellular Pel production. In addition, to better understand how c-di-GMP plays a role in VfmH-mediated Pel, complementation experiments using *vfmH*, *vfmH* ^R195A^, *vfmH* ^R279A^, or *vfmH* ^R344A^ cloned into plasmid pCL1920 controlled by the same *lac* promoter were conducted. Consistent with previous results (9, 16, 17), Δ*ecpC,* Δ*egcpB*, and Δ*vfmH* showed reduced extracellular Pel production relative to wild-type (WT) bacteria (Fig. 4A), and a similar reduction was also observed in Δ*vfmH*Δ*ecpC* and Δ*vfmH*Δ*egcpB*, respectively (Fig. 4A). Interestingly, complementation of *vfmH*, *vfmH* ^R195A^, *vfmH* ^R279A^, or *vfmH* ^R344A^ restored the Pel production in Δ*vfmH* to WT level, suggesting that the ability to bind c-di-GMP is not required for VfmH to control Pel under WT c-di-GMP-level conditions. By contrast, the expression of *vfmH* in Δ*vfmH*Δ*egcpB* did not complement the Pel activity but the expression of *vfmH* ^R195A^, *vfmH* ^R279A^, or *vfmH* ^R344A^ restored the mutant phenotype to WT levels (Fig. 4A). The *pelD* promoter activities were also determined in the above-mentioned strains (Fig. S1), and the results were consistent with the Pel activity assay (Fig. 4A). Since deletion of *egcpB* generates high c-di-GMP levels in the cell (9), our data suggest that high c-di-GMP inhibits the activity of VfmH to upregulate Pel in *D. dadantii*. Unlike Δ*egcpB*, our data showed that none of the *vfmH* constructs restored Pel production in Δ*vfmH*Δ*ecpC* (Fig. 4A), suggesting that an unknown mechanism is likely involved in EcpC-mediated Pel regulation independent of VfmH.

Since we found that complementation of Δ*vfmH* with *vfmH* ^R195A^, *vfmH* ^R279A^, or *vfmH* ^R344A^ recovered the Pel production to the WT levels, we next validated this observation by determining their virulence in potato tubers following the similar method as before (18). Δ*vfmH,* Δe*gcpB,* and Δ*vfmH*Δe*gcpB* showed reduced macerations due to the reduced Pel production (Fig. 4B). Complementation with *vfmH* restored the mutant phenotype to WT levels in Δ*vfmH* but not in Δ*vfmH*Δe*gcpB* (Fig. 4B), which is in agreement with the Pel activities (Fig. 4A). More importantly, the expression of *vfmH* ^R195A^, *vfmH* ^R279A^, or *vfmH* ^R344A^ recovered the virulence of Δ*vfmH* and Δ*vfmH*Δe*gcpB* to WT levels regardless of the c-di-GMP concentrations (Fig. 4B). Overall, these data further confirm that three Arg residues, Arg^195^, Arg^279^, and Arg^344^, in VfmH are essential for c-di-GMP-mediated suppression of virulence.

### VfmH interacts with CRP

To further understand the mechanism of VfmH-mediated Pel regulation, we performed a pull-down assay using VfmH as bait and the total *D. dadantii* proteins as prey to identify VfmH interacting partners. In this experiment, empty Ni-NTA resins with the WT cell lysate were used as a control for non-specific interactions. In total, 30 potential interacting proteins were identified using tandem mass spectrometry (Table S1; Fig. S2), by which CRP (Catabolite Repressor Protein) was identified.

**Fig 5:**
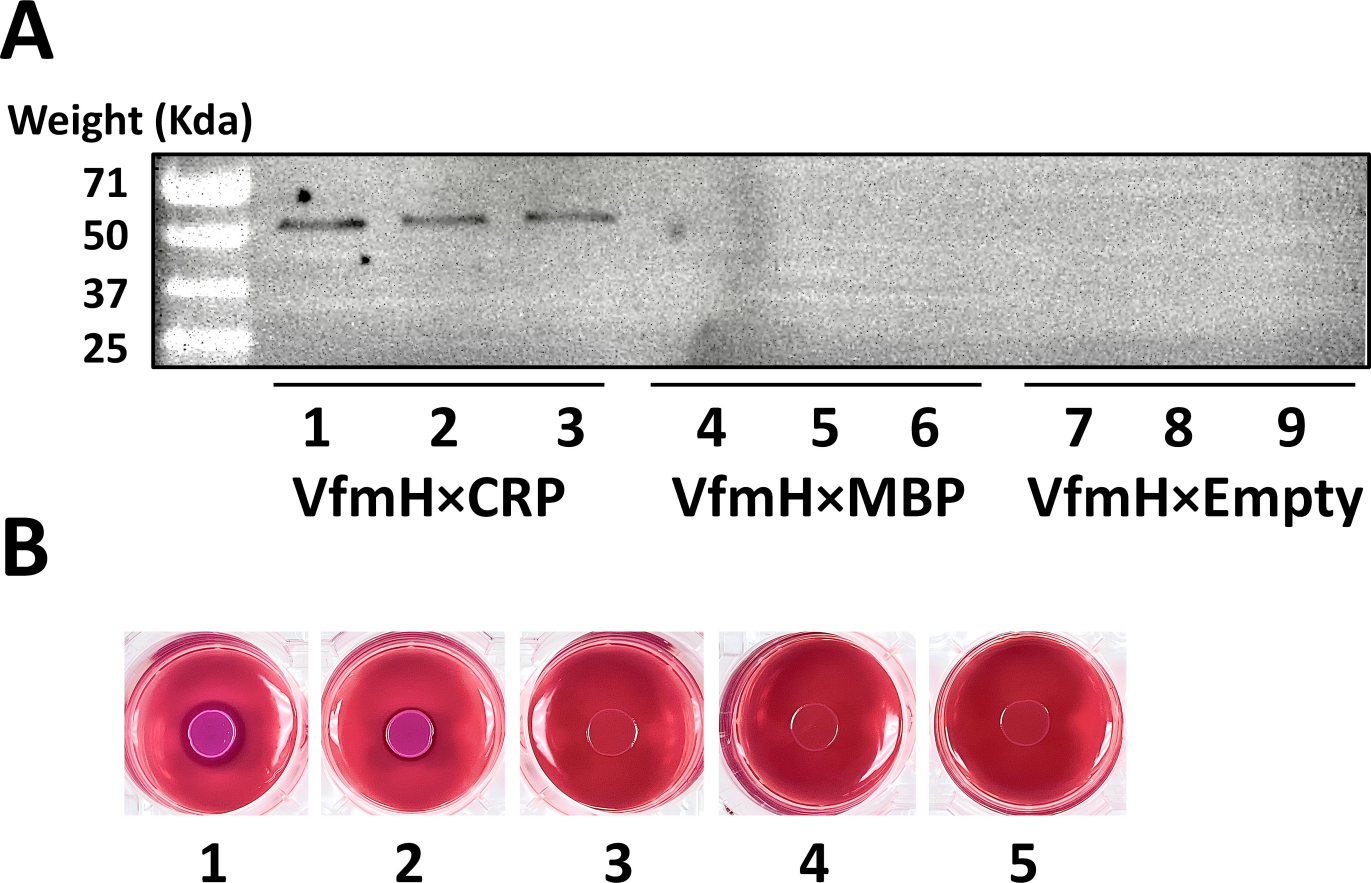
VfmH interacts with CRP. (**A**) VfmH was detected by western blot in MBP-CRP-coated amylose resin (lanes 1–3). Lanes 4–6 were loaded with samples eluted from MBP-coated amylose resin treated with histidine-tagged VfmH. Lanes 7–9 contain samples eluted from empty amylose resin treated with VfmH. No histidine-tagged VfmH was detected in lanes 4–9. (**B**) BACTH assay results as observed on MacConkey/Maltose agar plates. The first plate shows the positive control (dark red color, pKT25-zip/pUT18C-zip), and the second plate shows the interaction between VfmH and CRP (pKT25-*vfmH*/pUT18C-*crp*), a color similar to the positive control. Plates 3 (pKT25-*vfmH*/pUT18C), 4 (pKT25/pUT18C-*crp*), and 5 (pKT25/pUT18C) are negative controls that display a no color change of the colonies.

To confirm the specific interaction between VfmH and CRP, amylose resin coated with MBP-tagged CRP protein was used to pull down the histidine-tagged VfmH protein. We also included two negative controls, empty amylose resin with histidine-tagged VfmH protein and MBP-coated amylose resin with histidine-tagged VfmH protein, to evaluate non-specific interactions. Using an anti-histidine antibody, we observed a strong band of VfmH in the mixture of MBP-tagged CRP and histidine-tagged VfmH (Fig. 5A). By contrast, no bands were found in negative controls (Fig. 5A), suggesting that VfmH indeed binds CRP *in vitro*.

*In vivo* study of VfmH-CRP interaction was conducted using a bacterial adenylate cyclase two-hybrid system (BACTH) (29). Two plasmids pKT25 and pUT18C harboring *vfmH* and *crp* DNA fragments, respectively, were co-transformed into the *Escherichia coli* BTH101 strain. pKT25-zip and pUT18C-zip co-transformed strains were used as positive controls. *E. coli* strains containing pKT25-*vfmH* / pUT18C, pKT25/pUT18C-*crp*, or pKT25/pUT18C were used as negative controls. As expected, a qualitative analysis on MacConkey/Maltose plates showed dark red coloration of colonies in the positive control but the negative controls showed no color change. More importantly, the colonies containing pKT25-*vfmH*/pUT18C-*crp* showed dark red colonies similar to the positive control (Fig. 5B). Together, the above results demonstrate that VfmH and CRP interact with each other both *in vitro* and *in vivo*.

### VfmH involves CRP-cAMP to regulate *pelD* transcription

**Fig 6:**
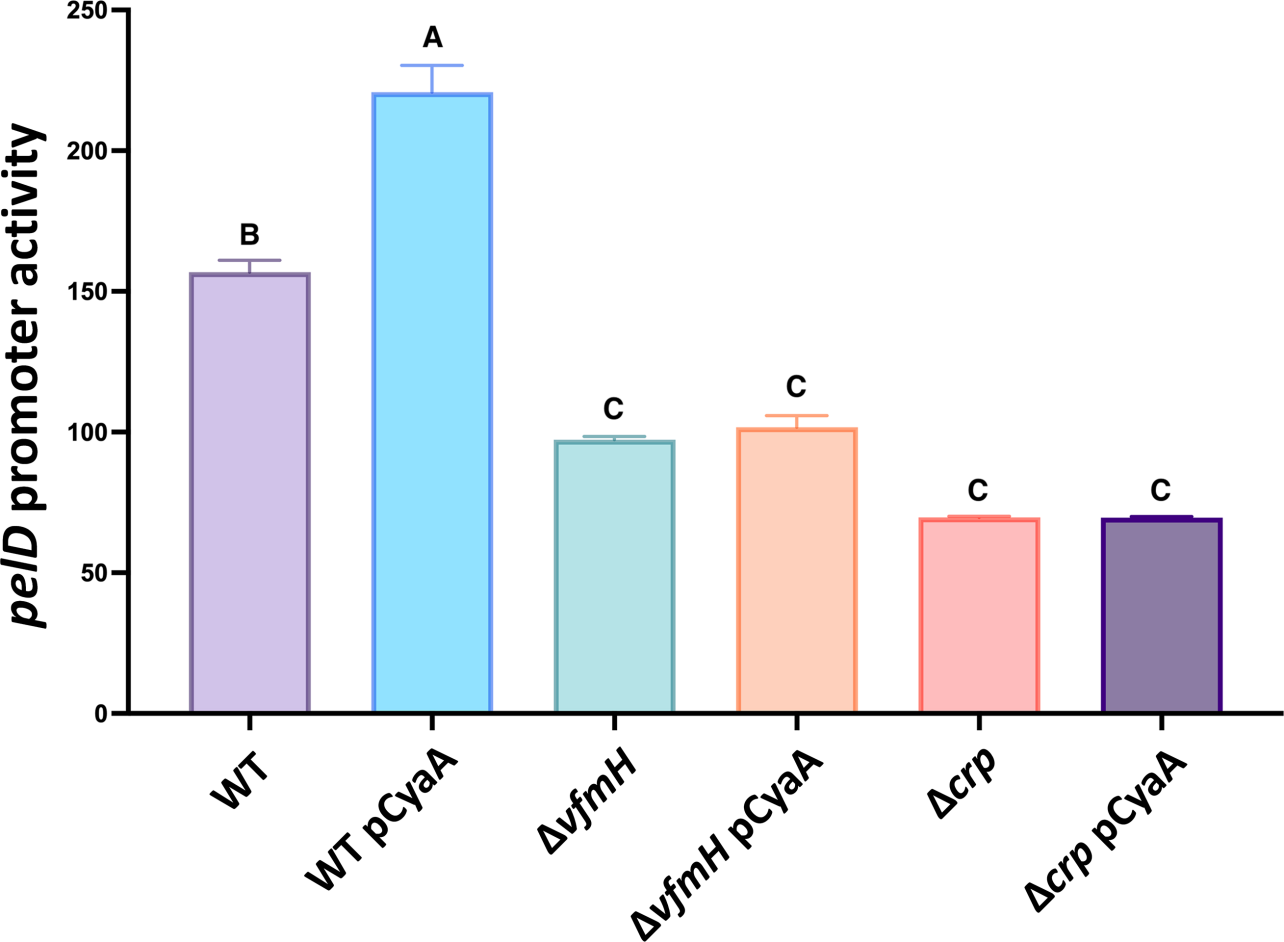
VfmH involves CRP-cAMP to regulate *pelD* transcription. (**A**) The transcriptional activity of *P_pelD_::gfp* in pPROBE-AT reporter plasmid was measured by flow cytometry. The *pelD* promoter activity was measured in WT, Δ*vfmH,* and Δ*crp* mutants with overexpression of *cyaA* in a low copy number plasmid pCL1920. Empty pCL1920 harboring strains were used as controls. Data were represented as mean fluorescence intensity; average GFP fluorescence intensity of total bacterial cells was examined. Values represent the mean of three independent experiments, and each experiment had triplicates. Error bars indicate the standard error of the mean. Different upper-case letters above the bars indicate statistical significance across different groups, whereas the same letters signify no statistical significance among the treatment groups in the M9 medium supplemented with 0.1% polygalacturonic acid for 24 h (*P* < 0.05) by one-way ANOVA.

Previous studies in *D. dadantii* demonstrated that CRP upregulates pectinolytic enzyme production (30) and the transcription of *pelD* and *pelE* genes is upregulated by the cAMP-CRP complex (31). Our data showed that VfmH upregulated Pel production (Fig. 4A) and interacted with CRP *in vitro* (Fig. 5), suggesting a crosstalk between these signaling pathways. Therefore, we checked the transcriptional activity of *P_pelD_::gfp* fusion (*pelD* promoter-GFP) in WT, Δ*vfmH,* and Δ*crp* with or without the overexpression of gene *cyaA* (encoding adenylate cyclase). An increased *pelD* transcription was observed in the WT strain overexpressing *cyaA* compared to that harboring an empty vector (Fig. 6). Previous studies have shown that CyaA synthesizes cAMP to induce CRP activity (32) and cAMP-CRP complex has been found to upregulate Pel in *D. dadantii* (31). Our data support these findings, as overexpression of *cyaA* did not affect *pelD* in *Δcrp* (Fig. 6). We also tested the *pelD* promoter activity in Δ*vfmH* overexpressing *crp*. Despite in *trans* expression of *crp* significantly increasing the *pelD* promoter activity in Δ*crp* to a level higher than the WT bacteria, it was not able to do so in Δ*vfmH*. Similarly, overexpression of *vfmH* in a *crp* deletion mutant did not restore its *pelD* promoter activity (Fig. S3). Taken together, these data agree that *pelD* transcription is upregulated by the cAMP-CRP complex as previously reported (31), and further suggest that VfmH plays a pivotal role in this regulation.

## DISCUSSION

VfmH has been previously characterized as the major response regulator of the Vfm QS that responds to the phosphate transfer from the sensory histidine kinase VfmI (16, 17). VfmH contains a typical AAA+ domain like many other bacterial enhancer binding proteins (bEBPs), such as FleQ from *P. aeruginosa* and FlrA from *Vibrio cholerae* (22, 23, 33). In this study, we functionally investigated several motifs within the AAA+ domain of VfmH, including Walker A and B motifs and three c-di-GMP interaction motifs. Our c-di-GMP binding assay demonstrated that VfmH binds to c-di-GMP *in vitro,* and we found that the conserved Arg residues at positions 195, 279, and 344 of the protein are responsible for this interaction. Substitution of these residues in VfmH with alanine abolished any interaction with c-di-GMP.

Bioinformatic analysis revealed that VfmH contains a CheY-family superdomain, which is closely related to swimming regulation in multiple bacteria and archaea (34 – 36). As expected, we found that VfmH upregulated swimming motility in *D. dadantii* (Fig. S4), and a similar observation has been reported in *D. zeae* (17). CheY plays an important role in the transmission of chemotaxis signals to the flagellar motors (35). In addition, c-di-GMP has been reported to repress swimming motility *via* allosterically binding to YcgR, by which YcgR alters the rotation of flagella (25, 37). Our data indicated that c-di-GMP has the ability to bind to VfmH and inhibit its function. This suggests that the two effectors, VfmH and YcgR, may play a role in the c-di-GMP-mediated swimming regulation in *D. dadantii*. Intriguingly, the VfmH homolog in *D. zeae* has been found to control swimming by modulating the expression of flagella biosynthesis genes (17). Consequently, whether VfmH controls the biosynthesis and/or rotation of flagella requires further investigation. In addition to c-di-GMP effectors, previous studies have identified PDEs, such as EGcpB and EcpC (9), as well as DGCs, such as GcpA and GcpL (10, 13), that are involved in the regulation of swimming. The mechanisms by which these c-di-GMP metabolic enzymes influence swimming through VfmH still need to be uncovered.

bEBPs, such as FleQ, are known to form higher oligomers, a process that is affected by c-di-GMP (22). Given the high homolog between VfmH and FleQ, we investigated the oligomerization pattern of VfmH and confirmed its ability to form dimers, trimers, and hexamers. However, the presence or absence of c-di-GMP or ATP did not affect the oligomerization pattern (Fig. S5). This suggests that c-di-GMP binding may not impact VfmH’s ability to form higher oligomers, as seen in other bEBPs (22). Conversely, it is plausible that c-di-GMP induces a conformational change in the oligomeric states of VfmH, which subsequently modifies its function. Our observation that c-di-GMP modestly inhibits the ATPase activity of VfmH supports this alternative explanation. For example, several bEBPs have been reported to initiate gene expression relying on their ATPase activity (38); although little is known about the regulatory function of ATPase activity in VfmH, our observation that the ATPase activity of VfmH was moderately inhibited by c-di-GMP may or may not affect VfmH to regulate gene transcription. Additional evidence is required to confirm this hypothesis; our findings, however, contribute insights into understanding the interactions between bEBPs and c-di-GMP molecules.

Although most bEBPs bind σ-factor (38) and VfmH contains σ-factor interaction motifs, we did not find any significant abundance of σ-factors in the pull-down assay. For example, RpoA, B, and C were identified (Table S1), but their abundance with respect to the negative control was not significant. This may be attributed to the fact that the abundance of most alternative σ-factors is stress related (39, 40). As a result, interactions between VfmH and σ-factors might only be detectable when bacterial cells are under stress. We also did not find σ−54 (also known as RpoN) in our pull-down assay, despite that VfmH contains a GAFTDA motif at positions 231–236, which resembles the well-characterized GAFTGA motif that enables the interaction between bEBPs and σ−54 (38, 41). Whether single residue substitution, such as G (glycine) to D (aspartic acid), of the GAFTGA motif accounts for the loss of interaction remains unknown. Another group of putative VfmH interactors that could not be detected in our pull-down assay is membrane-associated proteins. For example, VfmI, the histidine kinase in the VfmI/VfmH two-component system and a trans-membrane protein, was not discovered. Previously, we showed that SlyA, a transcriptional regulator, is involved in VfmE-mediated Pel regulation (18). SlyA was also detected in our pull-down assay, advocating further possible interactions with VfmH. It is noteworthy that several secretion proteins, including ABC family amino acid transporters and the SecA protein of the T2SS, were also pulled down with VfmH. This observation raises the possibility that VfmH may interact with the T2SS and facilitate the export of Pel through an as-yet-undetermined mechanism.

Previous studies demonstrated that VfmH upregulates Pel production in *Dickeya* (16, 17), and VfmE, which also binds c-di-GMP, fails to regulate Pel when intracellular c-di-GMP levels are high (18). Similarly, our data showed that under WT or low c-di-GMP-level conditions, VfmH positively regulated Pel; however, under high c-di-GMP-level conditions (e.g., Δ*egcpB* background), the binding between VfmH and c-di-GMP inhibited this regulation. Despite EGcpB and EcpC having been identified as PDEs and their deletion mutants causing elevated c-di-GMP levels to a similar degree (9, 25), our data showed that mutations of putative c-di-GMP interacting Arg residues in VfmH only restored its function in Δ*egcpB* but not in Δ*ecpC*. This is not surprising and, at least partially, could be explained by a temporal and spatial model that often occurs in c-di-GMP signaling (8, 42). In other words, differences in transcription, translation, and protein localization of these functionally similar PDEs might account for their diverse cellular outputs. In addition, EcpC could rely on a mechanism different from EGcpB in controlling Pel production, and such mechanisms do not require VfmH. In fact, a previous study aimed at c-di-GMP-mediated regulation of the type III secretion system concluded that EcpC has a stronger effect than EGcpB, and deletion of a c-di-GMP binding effector, YcgR, partially restored the phenotype in Δ*egcpB* and was unable to do so in Δ*ecpC* (25).

The interplays between QS and c-di-GMP signaling have been intensively studied in several bacterial species (43). For example, in *Vibrio cholerae*, Srivastava et al. found that VpsR, a transcriptional activator of QS and a bEBP, binds c-di-GMP. This interaction is essential for activating the QS pathway and promoting biofilm formation (44). Interestingly, we found that VfmH and a previously identified c-di-GMP effector VfmE both interact with c-di-GMP, and our data indicate that c-di-GMP negatively regulates their function. This is different from VpsR but similar to FleQ, whose function is inhibited by c-di-GMP (21 – 24). Under WT or low c-di-GMP-level conditions, we tested the *vfmE* promoter activity in Δ*vfmH* and found that VfmH is required for *vfmE* transcription (Fig. S6), which is in line with a previous study (16). However, the promoter activity of *vfmE* was not altered in Δ*crp* (Fig. S6), suggesting that the VfmH-Crp complex regulates Pel expression likely downstream of the VfmE-mediated mechanism.

**Fig 7:**
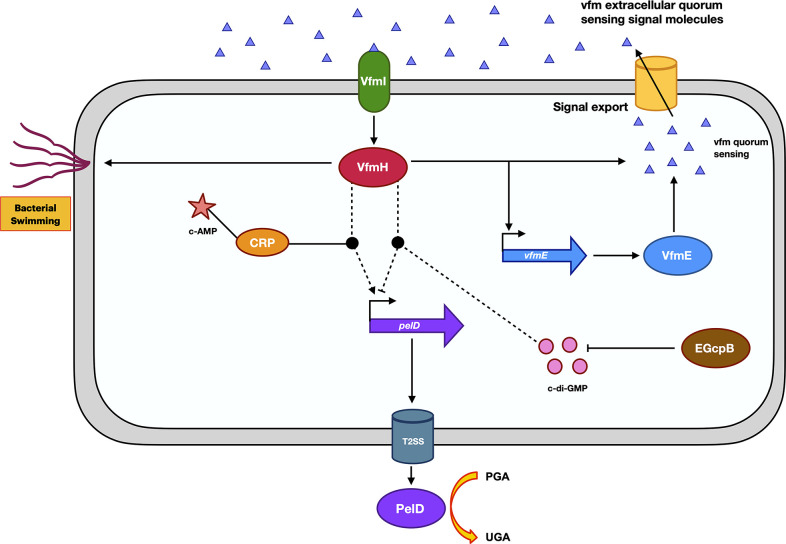
Regulatory cascade of VfmH. The regulation of different cellular phenotypes by VfmH. VfmH interacts with cAMP-CRP to positively regulate the *pelD* promoter. It upregulates swimming motility and Vfm QS system, including *vfmE*. The second messenger, c-di-GMP, is degraded by a PDE EGcpB, negatively impacting its concentration. Under WT (low) c-di-GMP condition, VfmH upregulates the transcription of *pelD* resulting in high virulence. But when the c-di-GMP level is high (Δ*egcpB*), VfmH binds to c-di-GMP and fails to control the transcription of the *pelD* gene culminating in low virulence. Regulatory pathways constructed from previous studies are shown with solid lines, and the regulatory pathways obtained from this study are shown with dotted lines.

In summary, we characterized the QS response regulator VfmH as a c-di-GMP binding effector protein. It acts as an activator under low c-di-GMP conditions; however, its function is inhibited when the c-di-GMP levels are high (Fig. 7). We also showed that VfmH hydrolyses ATP, a process that could be inhibited by the presence of c-di-GMP. We further demonstrated that VfmH interacts with c-di-GMP and CRP-cAMP to control Pel (Fig. 7). Previous study revealed interactions between CRP and c-di-GMP effectors. A recent investigation in *Shewanella putrefaciens* demonstrated that CRP directly interacts with a c-di-GMP effector, named BpfD. The binding of cAMP-CRP to BpfD enhances the ability of BpfD to interact with other proteins, consequently regulating biofilm formation (45). cAMP and c-di-GMP are two crucial bacterial signaling nucleotides that regulate bacterial behaviors and virulence. Our study uncovers the intricate crosstalk among the Vfm QS system, cAMP-CRP, and c-di-GMP signaling pathways. By uncovering these interactions, we can better comprehend how bacterial cells adapt their cellular behaviors and virulence in response to the changing environment.

## MATERIALS AND METHODS

### Bacterial strains, plasmids, primers, and media

Table S2 contains information about the bacterial strains and plasmids used in this study. All bacterial strains were stored at −80°C in 40% glycerol. Growth media used for *D. dadantii* strains are Luria–Bertani (LB) medium (1% tryptone, 0.5% yeast extract, and 1% NaCl), mannitol-glutamic acid (MG) medium (1% mannitol, 0.2% glutamic acid, 0.05% potassium phosphate monobasic, 0.02% NaCl, and 0.02% MgSO_4_), or low nutrient minimal medium (M9) supplemented with 2% glycerol at 28°C (46 – 48). *E. coli* strains were cultured in LB at 37°C. The following antibiotics were added to the medium at the concentrations mentioned: ampicillin (100 μg/mL), kanamycin (50 μg/mL), and streptomycin (50 μg/mL). The *D. dadantii* 3937 genome sequence can be obtained from the ASAP database (https://asap.ahabs.wisc.edu/asap/home.php). Primers used for PCR amplifications in this research are itemized in Table S3.

### Mutant construction, complementation, and overexpression

In-frame deletion of the *vfmH* gene was done by marker exchange mutagenesis (49). The Δ*vfmH,* Δ*vfmH*Δ*egcpB*, and Δ*vfmH*Δ*ecpC* mutants were constructed by the same procedure as described previously (18). To generate the complementation strains, the open-reading frame of *vfmH* was cloned into low copy number plasmid pCL1920 under the native *lac* promoter of the plasmid (Table S2). The resulting plasmids were PCR-confirmed and electroporated into the mutants. For overexpression of *cyaA,* the full length of the gene was PCR amplified from *E. coli* BW25113 (Keio collection, Japan) and cloned into pCL1920 under the *lac* promoter. The final constructed plasmids were electroporated into the cells.

### Green fluorescent protein (GFP) reporter plasmid construction and flow cytometry assay

The reporter plasmid pAT-*pelD* was previously constructed (9, 50, 51). Transcriptional activity was measured by quantification of the GFP intensity using flow cytometry (BD Biosciences, San Jose, CA, USA) as described (52). In brief, bacterial cells harboring the reporter plasmids were grown overnight in an LB medium and diluted 1:100 into an M9 medium supplemented with 0.1% polygalacturonic acid (PGA). Cells were harvested at 24 h and the GFP intensity was measured using a flow cytometer that corresponds to the promoter activity. In some cases, GFP intensity was measured using a BioTek (Agilent Technologies, Santa Clara, CA, USA) plate reader with excitation at 488 nm and emission at 535 nm. Mean fluorescence intensity was calculated based on the optical density at 600 nm values of bacterial cultures.

### Construction of point mutation

The open-reading frame of the *vfmH* gene was PCR amplified and cloned into the pCL1920 plasmid under the *lac* promoter using primers vfmH-F-HindIII and vfmH-R-BamHI (Table S3). To construct single amino acid substitution, three primer sets containing the point mutation, namely vfmH-R195A-1/vfmH-R195A-2, vfmH-R279A-1/vfmH-R279-2, and vfmH-R344A-1/vfmH-R344-2 along with vfmH-for-HindIII and vfmH-rev-BamHI (Table S3), were used to generate *vfmH* ^R195A^, *vfmH* ^R279A^, and *vfmH* ^R344A^, respectively, using pCL1920:*vfmH* ^WT^ as the template. The resulting fragment was joined by fusion PCR and cloned into pCL1920. The nucleotide(s) substitution was confirmed by DNA sequencing. The pCL1920:*vfmH* ^R195A^, pCL1920:*vfmH* ^R279A^, and pCL1920:*vfmH* ^R344A^ plasmids were used to complement the total Pel activity in Δ*vfmH,* Δ*vfmH*Δ*ecpC,* and Δ*vfmH*Δ*egcpB* mutants. The *vfmH* ^R195A^, *vfmH* ^R279A^, and *vfmH* ^R344A^ fragments were amplified from pCL1920:*vfmH* ^R195A^, pCL1920:*vfmH* ^R279A^, and pCL1920:*vfmH* ^R344A^ plasmids using the primers vfmH-for-NdeI and vfmH-rev-EcoRI (Table S3) and cloned into pET21b vector. The pET21b:*vfmH* ^R195A^, pET21b:*vfmH* ^R195A^, and pET21b:*vfmH* ^R195A^ plasmids were used for the overexpression of the resulting mutated protein to check c-di-GMP binding using ELISA.

### Protein expression and purification

Using the above-constructed pCL1920:*vfmH* plasmids as a template, the full length of *vfmH, vfmH* ^R195A^, *vfmH* ^R279A^, and *vfmH* ^R344A^ were cloned into the expression vector pET21-b under the inducible T7 promoter by primers vfmH-F-NdeI and vfmH-R-EcoRI (Table S3). The final plasmid constructs were validated by sequencing. The constructs expressing C-terminal 6X histidine-tagged *vfmH, vfmH* ^R195A^, *vfmH* ^R279A^, and *vfmH* ^R344A^ were transformed into *E. coli* BL21(DE3) cells for the overexpression of proteins, followed by purification. NEB Express^®^ *E. coli* cells (New England Biolabs, Ipswich, MA, USA) harboring empty pMAL-c6T plasmid that produces N-terminal 6Xhistidine-tagged MBP were used to purify MBP for negative controls in the ELISA experiment. In brief, to overexpress the fusion proteins, the T7 promoter of pET21b plasmid and *tac* promoter of pMAL-c6T was induced by the addition of isopropyl-thio-galactopyranoside (IPTG) at 0.1 mM final concentration, and the bacterial broth cultures were grown at 30°C for 4 h. The bacterial cells were centrifuged, and the pellet was resuspended in lysis buffer (50 mM NaH_2_PO_4_, 300 mM NaCl, pH 8.0) followed by cell lysis using sonication. To remove the cell debris from the lysate, samples were centrifuged at 15,000 rpm for 15 min. The soluble fraction thus obtained was analyzed by SDS-PAGE and western blot using HRP-conjugated Anti-His monoclonal antibody (Invitrogen, Waltham, MA, USA) to detect the presence of the recombinant proteins (Fig. S7). The soluble fractions containing the recombinant proteins were used for checking c-di-GMP binding in ELISA. For the purification of His-tagged proteins, the cell lysate was added to Ni-NTA resin for binding for 1 h. The protein-bound beads were washed three times with a wash buffer (50 mM NaH_2_PO_4_, 300 mM NaCl, and 20 mM Imidazole, pH 8.0) to remove any non-specific proteins from the resin. Then the protein was eluted with elution buffer (50 mM NaH_2_PO_4_, 300 mM NaCl, and 250 mM Imidazole, pH 8.0). C-terminal 6X-histidine-tagged YcgR from *D. dadantii* was purified following the same method used in a previous study (25). The purified proteins were analyzed by SDS-PAGE and western blot using Anti-His monoclonal primary antibody (Sigma-Aldrich, Burlington, MA, USA) and Anti-Mouse IgG-HRP conjugate secondary antibody (Southern Biotech, Birmingham, AL, USA) (Fig. S7). The purified histidine-tagged VfmH was used for checking ATPase activity.

### ATPase assay

ATPase activity assay was modified as described previously (22). Production of inorganic phosphates (Pi) due to the degradation of ATP was detected using a malachite green phosphate detection kit (R&D Systems, Minneapolis, MN, USA). Assays were performed in triplicates in a 1.5 mL microcentrifuge tube according to the manufacturer’s instructions. A standard curve with different concentrations of phosphate was constructed (Fig. S8). The reactions were conducted as follows: 200 µL total volume; 1 µM enzyme in ATPase buffer (25 mM HEPES pH 8.0, 300 mM NaCl buffer, 1 mM DTT, 10 mM KCl, 1 mM MgCl_2_, and 1 mM ATP) as per previous study (22). For the competition, 100 µM c-di-GMP was added to the reaction. The samples were incubated in a 28°C incubator. The reactions were monitored at 15-min intervals up to 60 min. Malachite A reagent was added and incubated for 10 min, followed by the addition of Malachite B reagent and incubation for 20 min at room temperature. The color intensities were detected in a 96-well plate and a microplate reader at 620 nm. The data were represented as a curve showing nmol Pi/μg protein (53). No ATPase activity was observed while using cell lysate from *E. coli* BL21 with empty vector pET21b purified over Ni-NTA. The rate of Pi generated from ATP by VfmH was determined by the following formula: (nMol Pi at time 60 min − nMol Pi at time 0 min)/1 µg VfmH/60 mins.

### Pull-down assay

To identify the interacting partners of VfmH, an *in vitro* pull-down assay was performed, modified from the method described previously (54). In brief, histidine-tagged VfmH was overexpressed in BL21(DE3) cells for 4 h, and the cells were collected by centrifugation. The cell pellets were resuspended in a lysis buffer and sonicated. The cell debris was removed by centrifugation, and the buffer containing His-tagged VfmH was used to coat Ni-NTA resins for 1 h. WT *D. dadantii* cells were collected from an overnight culture, and cells were collected by centrifugation. The cells were resuspended in a lysis buffer and sonicated. The cell debris was removed, and the buffer containing a mixture of *D. dadantii* proteins was added to histidine-tagged VfmH-coated Ni-NTA resins and incubated for 6 h. The resins were washed three times with wash buffer and eluted by boiling the resins in SDS elution buffer (1 M Tris-HCL, 0.5 M EDTA, and 10% SDS, pH 8.0) at 97°C for 10 min. The eluted proteins were analyzed by SDS-PAGE and silver staining, followed by identification with MALDI-tandem mass spectrometry (University of Wisconsin-Madison). In this experiment, cell lysate from WT *D. dadantii* cells was added to Ni-NTA resins without His-tagged VfmH as a control for background binding.

To confirm the interaction between CRP and VfmH, an MBP-tagged variant of CRP was constructed and cloned into a pET21-b vector. MBP-CRP was then overexpressed in *E. coli* BL21(DE3) cells for 4 h, and cells were collected by centrifugation. The cells were resuspended in MBP column binding buffer (20 mM Tris-HCl, 200 mM NaCl, 1 mM DTT, pH 7.5) followed by sonication. The cell debris was removed by centrifugation and the buffer containing MBP-tagged CRP was used to coat amylose resins for 1 h. *E. coli* cells expressing His-tagged VfmH were collected from a 4-h culture, and cells were collected by centrifugation. The cells were resuspended in a lysis buffer and sonicated. The cell debris was removed and the buffer containing histidine-tagged VfmH protein was added to MBP-CRP-coated amylose resins and incubated for 6 h. The resins were washed three times with MBP column binding buffer and eluted with MBP elution buffer (20 mM Tris-HCl, 200 mM NaCl, 1 mM DTT, 10 mM Maltose, pH 7.5). The histidine-tagged VfmH was detected by western blot using an Anti-His monoclonal primary antibody (Sigma-Aldrich, Burlington, MA, USA) and Anti-Mouse IgG-HRP conjugate secondary antibody (Southern Biotech, Birmingham, AL, USA). To confirm interactions, His-tagged VfmH was added to either amylose beads coated with MBP protein or untreated amylose resins. The expression of histidine-tagged MBP was detected by western blot and CRP-MBP was analyzed by SDS-PAGE and Coomassie staining before performing the assay (Fig. S7).

### Bacterial two-hybrid interaction assay

The interaction of VfmH and CRP was confirmed *in vivo* by a bacterial adenylate cyclase two-hybrid system (Euromedex, Souffelweyersheim, France). The assay was performed according to the manufacturer’s instructions. Briefly, *vfmH* and *crp* open-reading frames were cloned into two plasmids, pKT25 and pUT18C, from the *D. dadantii* 3937 genome. The resulting constructs were co-transformed into the *E. coli* BTH101 strain. Strain with co-transformed pKT25-zip/pUT18C-zip plasmids was kept as a positive control. Strains harboring pKT25-*vfmH*/pUT18C, pKT25/pUT18C-*crp*, and pKT25/pUT18C were used as negative controls. The colonies were selected on MacConkey agar plates (Difco, Franklin Lakes, NJ, USA) supplemented with 1% maltose, 100 µg/mL ampicillin, and 50 µg/mL kanamycin. Single colonies were grown overnight on LB with antibiotics, washed three times with sterile water, and 10 µL was spotted on MacConkey/maltose agar plates supplemented with 0.5 mM IPTG, ampicillin, and kanamycin and incubated at 30°C for 48 h. The colors of the colonies containing pKT25-*vfmH*/pUT18C-*crp* were observed for qualitative estimation of complementation of the phenotype that indicates interaction between the proteins.

### Pel activity assay

Pel activity was determined using spectrometry as described previously (18, 55). Overnight bacterial cultures in LB broth were inoculated 1:100 into fresh LB broth supplemented with 0.1% PGA at 28°C for 16 h. OD_600_ values of bacterial cultures were measured. Culture supernatant was collected by centrifugation at 15,000 rpm for 2 min. Ten microliter of supernatant was added into 990 µL of reaction buffer [0.05% PGA, 0.1 M Tris-HCl (pH 8.5), and 0.1 mM CaCl_2_, prewarmed to 30°C]. The Pel activity was measured at 230 nm (A_230_) for 3  min. The rate was calculated by the following: one unit of Pel activity is equal to an increase of 1  ×  10^−3^ optical density at 230 nm (A_230_) in 1  min. The cell density (OD_600_) of each bacterial culture was measured. The final Pel activity was represented as A_230_/OD_600_, i.e., total extracellular Pel activity normalized by the cell density of the culture.

### Potato infection assay

Potato infection assay was performed according to the experiments described previously (9, 18) using russet potato (*Solanum tuberosum*). Briefly, 1  cm thick slices of the potatoes were injected with 50  µL bacterial suspension at 10^6^ CFU mL^−1^ using a syringe. Each bacterial strain was infected in three different potato slices (triplicate). The potato slices inoculated with the bacteria were kept in a 28°C incubator with 100% relative humidity. The necrosis of potato tissue was measured by scooping out the soft necrotic tissue and measuring the weight of each sample. The loss of weight represents the amount of macerated tissue.

### Statistical analysis

Statistical analysis was done using one-way ANOVA with PRISM 9 software (GraphPad Software, San Diego, CA, USA) together with Tukey’s multiple comparisons tests. The significance was checked at a 95% confidence interval (*P* < 0.05). Data in the graphs represent means ± standard error of mean or standard deviation wherever indicated.

## SUPPLEMENTAL MATERIAL

Supplemental figures and tables spectrum.01537-23-s0001.pdf Table S1 to S3; Figure S1 to S8

ASM does not own the copyrights to Supplemental Material that may be linked to, or accessed through, an article. The authors have granted ASM a non-exclusive, world-wide license to publish the Supplemental Material files. Please contact the corresponding author directly for reuse.
