# Supplementary material for: Dissecting the molecular dance: c-di-GMP, cAMP-CRP, and VfmH collaboration in pectate lyase regulation for Dickeya dadantii—unveiling the soft rot pathogen’s strategy
Source: Microbiol Spectr. 2023 Oct 9;11(6):e01537-23. doi: 10.1128/spectrum.01537-23 (PMC10714721; doi:10.1128/spectrum.01537-23)
Supplement: Supplemental figures and tables — Table S1 to S3; Figure S1 to S8 [file spectrum.01537-23-s0001.pdf]

Table S1. Putative VfmH-interacting proteins.

Identified Proteins	Gene ID	Molecular weight	Abundance of proteins in MS spectra		
			Control spectral count [a]	VfmH spectral count [b]	Ratio (VfmH spectral count/ Control spectral count)
VfmH histidine-tagged	*vfmH*	55 kDa	0	68	
Maltoporin	*ganL*	46 kDa	1	16	16
Catabolite activator protein	*crp*	24 kDa	2	21	10.5
Phosphoglycerate kinase	*pgk*	41 kDa	1	9	9
6-phosphogluconate dehydrogenase	*gnd*	51 kDa	1	8	8
Protein translocase subunit SecA	*secA*	102 kDa	1	8	8
Putrescine-binding periplasmic protein	*potF*	41 kDa	1	6	6
Fructose-bisphosphate aldolase	*fbaA*	39 kDa	1	6	6
ATP-dependent protease ATPase subunit HslU	*hslU*	50 kDa	2	11	5.5
CTP synthase	*pyrG*	60 kDa	2	10	5
6-phosphogluconolactonase	*ybhE*	36 kDa	1	5	5
4-hydroxy-tetrahydrodipicolinate reductase	*dapB*	29 kDa	1	5	5
Serine hydroxymethyltransferase	*glyA*	45 kDa	1	5	5
Outer membrane porin 1a (IabF)	*ompF*	40 kDa	3	13	4.33
Aspartate ammonia-lyase	*aspA*	52 kDa	6	25	4.17
NADH-quinone oxidoreductase	*nuoG*	99 kDa	2	8	4
ATP synthase subunit beta	*atpD*	50 kDa	1	4	4
Malate dehydrogenase	*mdh*	32 kDa	2	7	3.5
NADP-linked malic enzyme	*maeB*	82 kDa	2	7	3.5
Transcription termination factor Rho	*rho*	47 kDa	2	7	3.5
Citrate synthase	*gltA*	48 kDa	5	16	3.2
Periplasmic-binding component of an ABC superfamily	*gltI*	34 kDa	12	37	3.08

glutamate/aspartate transporter					
GMP synthase	*guaA*	58 kDa	1	3	3
Transcriptional regulator SlyA	*slyA*	16 kDa	1	2	2
ABC-type transporter, periplasmic component	*Dda3937_03671*	61 kDa	1	2	2
DNA-directed RNA polymerase subunit alpha	*rpoA*	37 kDa	8	15	1.87
DNA-directed RNA polymerase subunit beta	*rpoB*	151 kDa	24	41	1.71
DNA-directed RNA polymerase subunit beta	*rpoC*	155 kDa	24	36	1.5

[a]Abundance of proteins detected from empty Ni-NTA resins.
[b]Abundance of proteins detected from histidine-tagged VfmH coated Ni-NTA resins using MALDI-TOF/TOF analysis.

Table S2. Strains and plasmids used in this study.

Strains and plasmids	Relevant characteristics[a]	Reference or source
Dickeya dadantii		
3937	Wild-type	Lab stock
Δ*ecpC*	Δ*ecpC*, ABF-0020364 deletion mutant	(1)
Δ*vfmH*	Δ*vfmH*::*Km;* Km^r, ABF- 0016069 deletion mutant	This study
Δ*egcpB*	Δ*egcpB*, ABF-0020123 deletion mutant	(1)
Δ*vfmH*Δ*ecpC*	Δ*vfmH*Δ*ecpC*::*Km;* Km^r, ABF-0016069 and ABF-0020364 double deletion mutant	This study
Δ*vfmH*Δ*egcpB*	Δ*vfmH*Δ*egcpB*::*Km;* Km^r, ABF-0016069 and ABF-0020123 double deletion mutant	This study
Δ*crp*	Δ*crp*::*Cm;* Cm^r, ABF-0015482 deletion mutant	(2)
Escherichia coli		
DH5α	*supE*44 Δ*lac*U169 (*ϕ*80*lacZ*ΔM15) *hsdR*17 *recA*1 *endA*1 *gyrA*96 *thi*-1 *relA*1	Lab stock
S17-1 λpir	λ(pir) *hsdR* pro *thi*; chromosomally integrated RP4-2 Tc::Mu Km::Tn7	Lab stock
BL21(DE3)	B F$^-$ *ompT gal dcm lon hsdS*$_B$($r_B^- m_B^-$) λ(DE3 [*lacI lacUV5-T7p07 ind1 sam7 nin5*]) [*malB*$^+$]$_{K\text{-}12}$(λ^S)	Lab stock
BTH101	F$^-$, *cya-99*, *araD139*, *galE15*, *galK16*, *rpsL1* (Str^r), *hsdR2*, *mcrA1*, *mcrB1*.	Euromedex
**Plasmids**		
pKD4	Template plasmid for kanamycin cassette, Km^r	(3)
pWM91	Sucrose-based counter-selectable plasmid, Ap^r	(4)
pWM91-*vfmH*	pWM91 harboring flanking regions of *vfmH* with kanamycin cassette in between, Km^r, Ap^r	This study
pET21b	Overexpression and purification vector, Ap^r	Novagen
pET21b- *ycgR*$_{3937}$	Overexpression of *ycgR*$_{3937}$ in expression vector	(5)
pET21b-*vfmH*	Overexpression of *vfmH* in expression vector, Ap^r	This study
pET21b-*malE-crp*	Overexpression of MBP tagged CRP in expression vector, Ap^r	This study
pCL1920	Low copy number plasmid, lac promoter, Sp^r	(6)
pCL-*vfmH*	*vfmH* cloned in pCL1920 under *lac* promoter, Sp^r	This study
pCL- *vfmH*^{R195A}	*vfmH*^{R195A} cloned in pCL1920 under *lac* promoter, Sp^r	This study
pCL- *vfmH*^{R279A}	*vfmH*^{R279A} cloned in pCL1920 under *lac* promoter, Sp^r	This study
pCL- *vfmH*^{R344A}	*vfmH*^{R344A} cloned in pCL1920 under *lac* promoter, Sp^r	This study
pCL-*cyaA*	*cyaA* cloned in pCL1920 under *lac* promoter, Sp^r	This study
pCL-*crp*	*crp* cloned in pCL1920 under native *crp* promoter, Sp^r	This study
pPROBE-AT	Promoter-probe vector, promoter-less *gfp*, Ap^r	(7)
pAT-*pelD*	pPROBE-AT containing *pelD* promoter-*gfp* transcriptional fusion, Ap^r	(8)
pAT-*vfmE*	pPROBE-AT containing *vfmE* promoter-*gfp* transcriptional fusion, Ap^r	(9)
pFLP2	Plasmid containing the *flp* (flippase) gene, Amp^r	Lab stock
pKT25	Overexpression plasmid for bacterial two-hybrid assay, expressing the T25 fusion fragment, Km^r	Euromedex
pUT18C	Overexpression plasmid for bacterial two-hybrid assay, expressing T18 fusion fragment, Ap^r	Euromedex
pKT25-zip	Derivative of pKT25, the leucine zipper of GCN4 is genetically fused in frame to the T25 fragment, Km^r	Euromedex
pUT18C-zip	Derivative of pUT18C, the leucine zipper of GCN4 is genetically fused in frame to the T25 fragment, Ap^r	Euromedex
pKT25-*vfmH*	*vfmH* gene cloned into pKT25 plasmid, in-frame with T25 fragment, Km^r	This study

pUT18C-*crp*	*crp* gene cloned into pUT18C plasmid, in-frame with T18 fragment, Ap^r	This study

[a]Ap^r, ampicillin resistance; Km^r, kanamycin resistance; Sp^r, streptomycin resistance; Cm^r, chloramphenicol resistance

Table S3. Primers used in this study.

Primers	Sequences (5’-3’)	Use
vfmH-A-XhoI<br>*vfmH*-B<br>*vfmH*-C<br>*vfmH*-D-NotI	AATACTCGAGAGCGGATTCAGGAGATAGGT<br>GAAGCAGCTCCAGCCTACACCTATGTTGCTTATCCGCCATA<br>CTAAGGAGGATATTCATATGACTCGACTCCTCCTTTGAGC<br>AATATTATGCGGCCGCGCCCGAACTCCGCCGCCGGT	*vfmH* deletion
vfmH-R195A-1<br>*vfmH*-R195A-2<br>*vfmH*-R279A-1<br>*vfmH*-R279A-2<br>*vfmH*-R344A-1<br>*vfmH*-R344A-2	CCGGCAAAAGCCTGCTGGCCGCGGTCATTCATCAGCTTTCTCCGCGC<br>GCGCGGAGAAAGCTGATGAATGACCGCGGCCAGCAGGCTTTTGCCGG<br>GCGTATTCTGGAAGAACGCTGTTTTGAGGCGGTTGGCTCGTCCCGTACGCT<br>AGCGTACGGGACGAGCCAACCGCCTCAAAACAGCGTTCTTCCAGAATACGC<br>GCCGCTGGCGTTGAATTTCATTGATGCCTATGCCAGAAAATACAATAAGCCGGCGC<br>GCGCCGGCTTATTGTATTTTCTGGCATAGGCATCAATGAAATTCAACGCCAGCGGC	*vfmH* site-directed mutant
vfmH-for-NdeI<br>*vfmH*-rev-EcoRI	TATCGTCGACGGATCCATGAGCTTGCAGAACACCTACG<br>GTTTTATTTGAAGCTTATTACAGAGGTTCTGGATATTATCCAGCAG	*vfmH* overexpression
vfmH-for-HindIII<br>*vfmH*-rev-BamHI	TGATTACGCCAAGCTTAATGGCGGATAAGCAACATATCCTGATTGTTGATGAT<br>CGGTACCCGGGGATCCTTACTCATGGTTAATCTTGTATTTTTCCAGTCGTCG	*vfmH* complementation
vfmH-F-BamHI<br>*vfmH*-R-KpnI	CGACTCTAGAGGATCCAATGGCGGATAAGCAACATATCCTGATTGT<br>TTCTTAGTTACTTAGGTACCTTACTCATGGTTAATCTTGTATTTTTCCAGTCGTCGGT	*vfmH* overexpression in pKT25 plasmid
P1<br>P2	GCGATTGTGTAGGCTGGAGCTGCTTC<br>GCTGACATGGGAATTAGCCATGGTCC	Kanamycin cassette amplification from pKD4 plasmid
vfmH-F-SalI<br>*vfmH*-R-HindIII	ATCCTCTAGAGTCGACTTTACACTTTATGCTTCCGGCTCG<br>TGGGGATCGGAAGCTTTTACTCATGGTTAATCTTGTATTTTTCCAGTCGTCGG	*vfmH* gene under lac promoter amplification from pCL1920-*vfmH*
MBP-F-NdeI<br>MBP-R-EcorI	AAGGAGATATACATATGGAAGAAGGTAAACTGGTAATCTGGATTAACGG<br>GACGGAGCTCGAATTCAGTCTGCGCGTCTTTCAGG	MBP overexpression in pET21b vector, amplified from pMAL-c6t
crp-F-SalI<br>*crp*-R-HindIII	TTCGAGCTCCGTCGACATATGGTTCTCGGCAAACCGC<br>GTGCGGCCGCAAGCTTTCAGCGGGTGCCGTATACC	Overexpression of *crp* in frame with MBP in pET21b vector
crp-F-BamHI<br>*crp*-R-EcoRI	CGACTCTAGAGGATCCAATGGTTCTCGGCAAACCGC<br>TTATATCGATGAATTCGCGGGTGCCGTATACCAC	*crp* overexpression in pUT18C plasmid
crp-F-1920<br>*crp*-R-1920	gactctagaggatccccCACCGCTTATGTATAAAAAAGTCTGATAATCAGAGC<br>tcgagctcggtacccTTTGATCCGGTGGATGATAATCAGCG	*crp* overexpression in pCL1920 plasmid
cyaA-F-SalI<br>*cyaA*-R-KpnI	ATGCCTGCAGGTCGACTTGTACCTCTATATTGAGACTCTGAAACAGAGACT<br>GTGAATTCGAGCTCGGTACCTCACGAAAAATATTGCTGTAATAGCGGC	*cyaA* overexpression under lac promoter in pCL1920 vector

Underlined letters represent the sequence of restriction enzymes used for cloning. For site-directed mutagenesis, underlined letters represent the change from arginine to alanine. The lower letters represent recombinant sequences to the plasmid pCL1920.

Figure S1. *pelD* promoter activity in various *D. dadantii* strains.

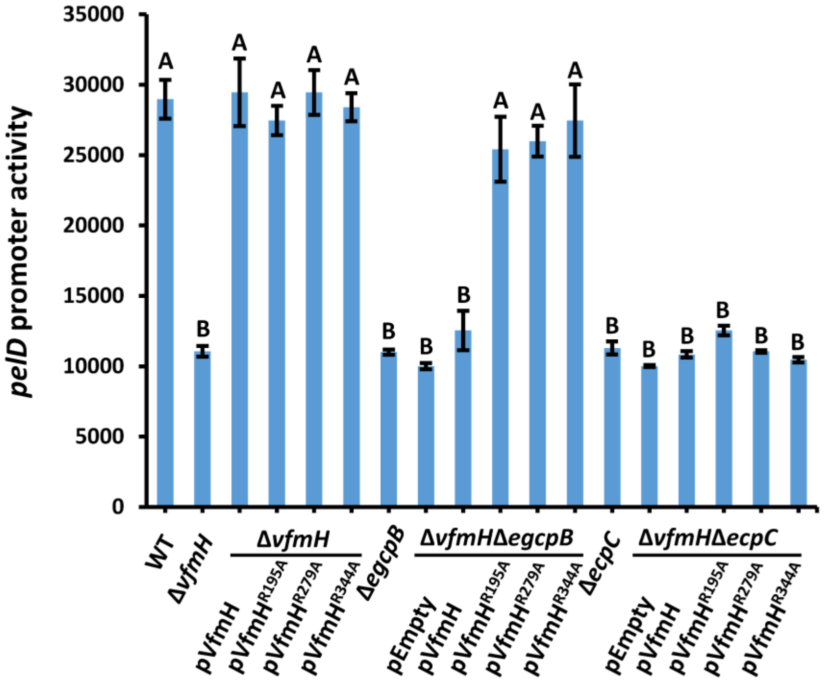

The transcriptional activity of P_{pelD}::*gfp* in pPROBE-AT reporter plasmid was measured in wild-type (WT) *D. dadantii*, Δ*vfmH*, Δ*egcpB*, and Δ*ecpC* strains, and Δ*vfmH*, Δ*vfmH*Δ*egcpB*, and Δ*vfmH*Δ*ecpC* bearing low copy number plasmid pCL1920, pCL1920:*vfmH*, pCL1920:*vfmH*^{R195A}, pCL1920:*vfmH*^{R279A}, or pCL1920:*vfmH*^{R344A}. Values represent the mean fluorescence intensity. Three replicates per experiment and three experiments were conducted. The essay was performed using a plate reader. Error bars indicate the standard error of the mean. Different upper-case letters above the bars indicate statistical significance ($P < 0.05$) by one-way ANOVA.

Figure S2. Silver stain showing pulled down proteins with Histidine tagged-VfmH.

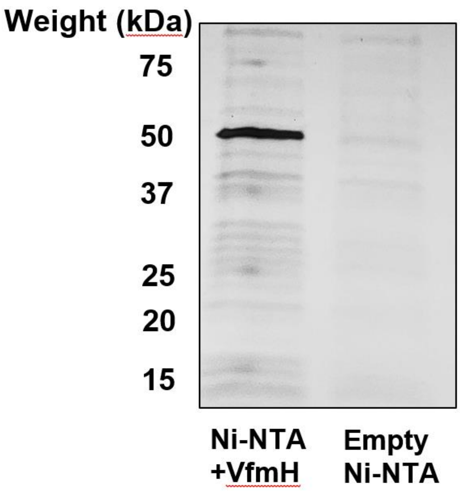

Proteins pulled down with C-terminal histidine-tagged variant of VfmH was analyzed by SDS PAGE and silver staining (Pierce Silver staining kit, Thermofisher). Lane 1 shows the proteins that were pulled down by this process. The strongest band represents histidine tagged VfmH protein. Lane 2 shows the proteins that non-specifically bind to Ni-NTA resins (background control).

Figure S3. VfmH-Crp complex is required for *pelD* transcription.

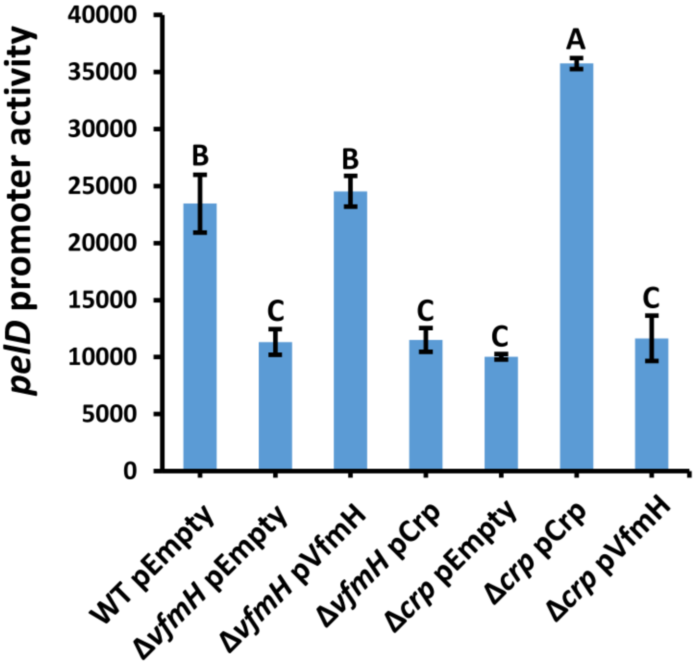

The transcriptional activity of P_{pelD}::*gfp* in pPROBE-AT reporter plasmid was measured in wild-type (WT) *D. dadantii*, Δ*vfmH*, and Δ*crp* bearing an empty vector pCL1920, and Δ*vfmH* or Δ*crp* bearing pCL1920:*vfmH* or pCL1920:*crp*. Values represent the mean fluorescence intensity. Three replicates per experiment and three experiments were conducted. The essay was performed using a plate reader. Error bars indicate the standard error of the mean. Different upper-case letters above the bars indicate statistical significance ($P < 0.05$) by one-way ANOVA.

Figure S4. VfmH upregulates swimming motility.

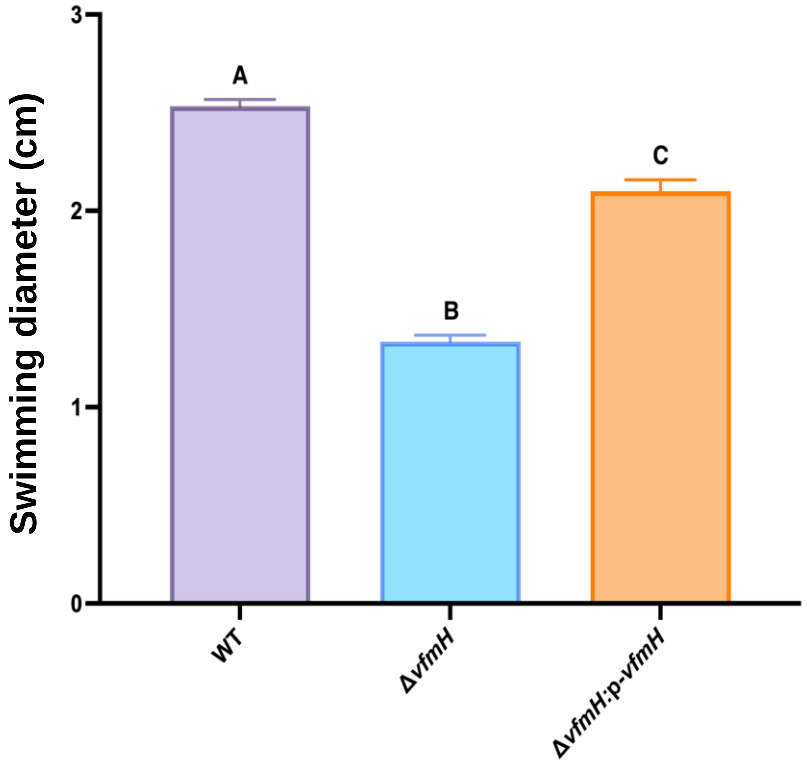

The swimming motility of WT and Δ*vfmH* was measured on the soft agar plate. Complementation of the phenotype in Δ*vfmH* was done with pCL1920:*vfmH.* Values represent the mean of three independent experiments, and each experiment had triplicates. Error bars indicate the standard error of the mean. Different upper-case letters above the bars indicate statistical significance across different groups, whereas the same letters signify no statistical significance among the treatment groups for 12 h ($P < 0.05$) by one-way ANOVA.

Figure S5. Oligomerization pattern of VfmH.

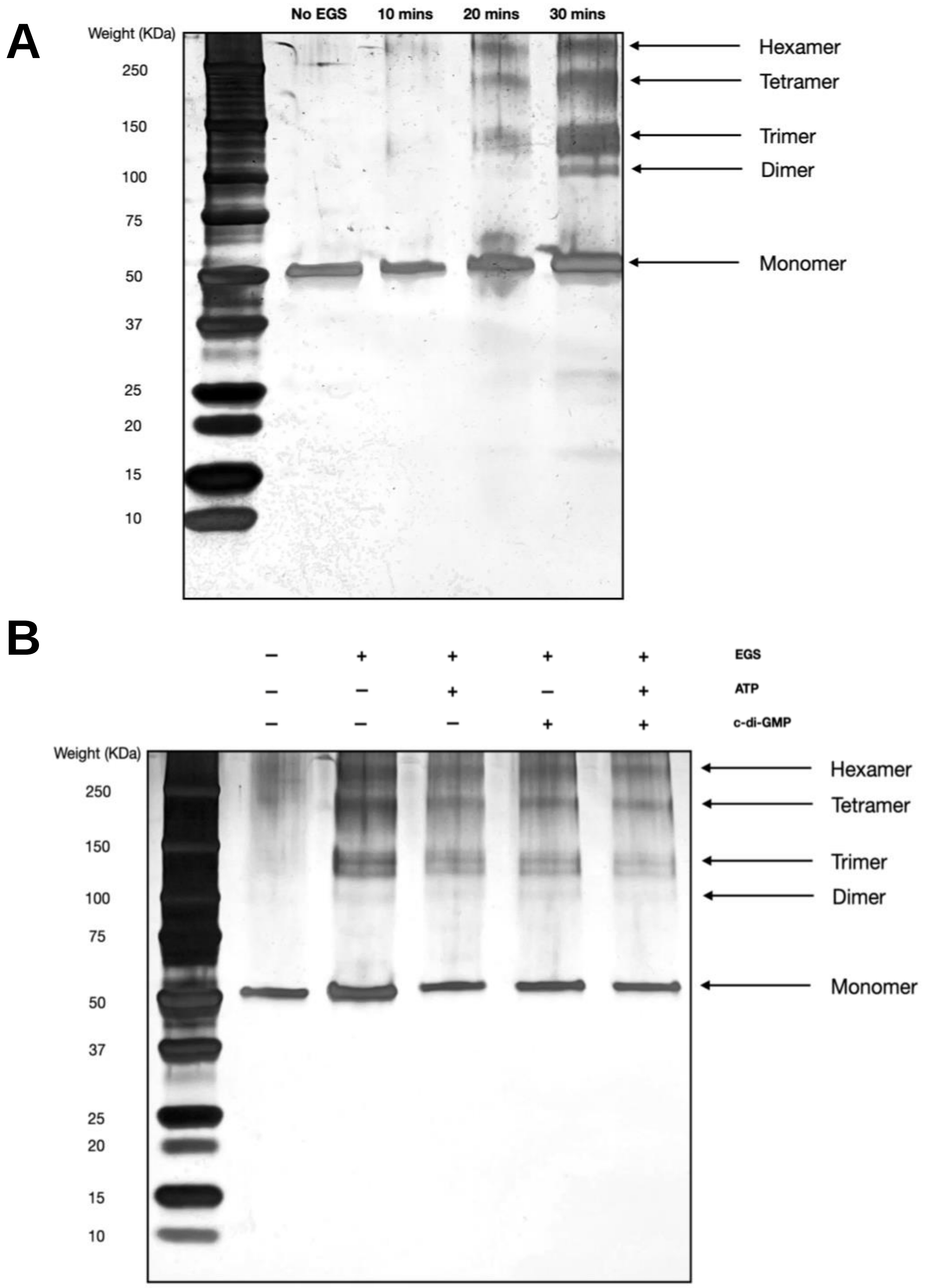

The oligomerization of VfmH was studied following the procedure described previously (10). (A) Oligomerization of VfmH over time in presence of Ethylene glycol bis-(N-hydroxy succinimidyl succinate) [EGS] crosslinker. VfmH forms higher oligomers typically like bacterial enhancer binding proteins. (B) Oligomerization of VfmH in the presence or absence of nucleotides (ATP and c-di-GMP). No observable reduction of oligomers of VfmH was noticed in the presence of ATP or c-di-GMP, suggesting it possibly undergoes a conformational change to alter its regulation properties when bound to the nucleotides. The results were detected by SDS-PAGE and Pierce silver staining.

Figure S6. VfmH but not Crp is required for *vfmE* transcription.

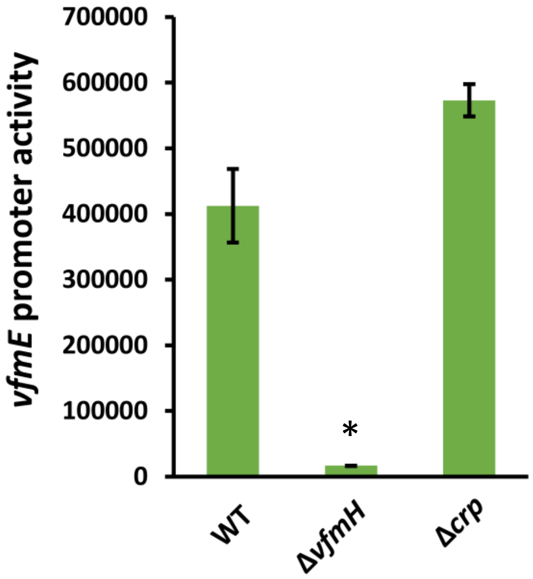

The transcriptional activity of P_{vfmE}::*gfp* in pPROBE-AT reporter plasmid was measured in wild-type (WT) *D. dadantii*, Δ*vfmH*, and Δ*crp*. Values represent the mean fluorescence intensity. Three replicates per experiment and three experiments were conducted. The essay was performed using a plate reader. Error bars indicate the standard error of the mean. Asterisks indicate statistically significant differences of the means ($P < 0.05$ by Student's *t*-test)

Figure S7. Overexpressed recombinant proteins detection by western blot and Coomassie staining.

A

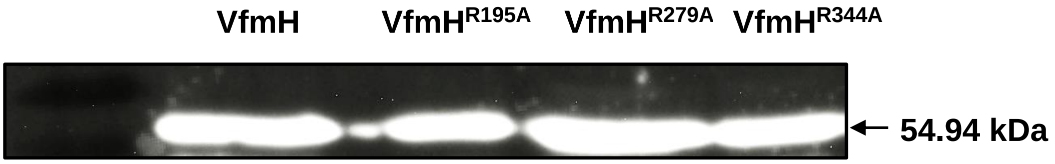

B

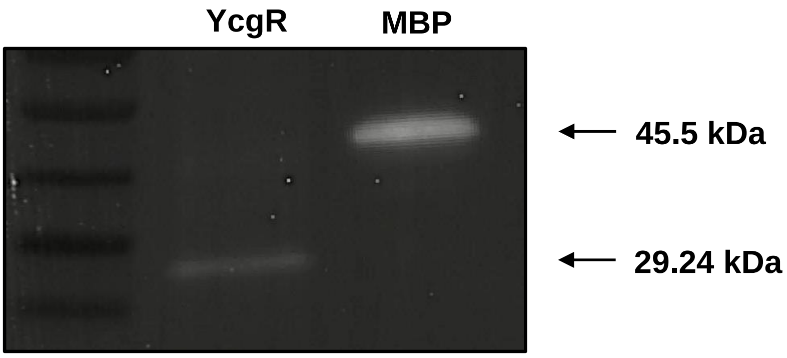

C

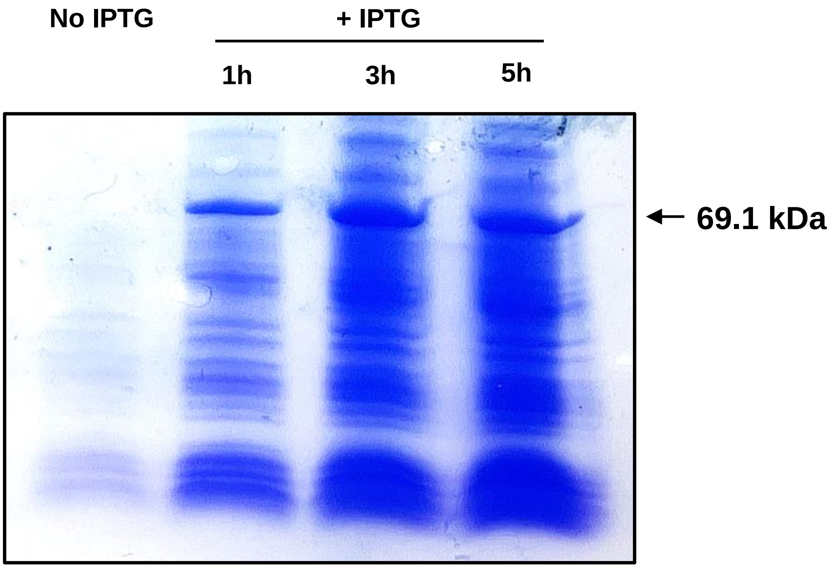

(A) Histidine-tagged VfmH, VfmH^{R195A}, VfmH^{R279A}, and VfmH^{R344A} proteins detected by mouse anti-His monoclonal antibody (Sigma Aldrich) and goat anti-mouse HRP conjugate secondary antibody (Southern Biotech). Lane 1 represents VfmH, Lane 2 represents VfmH^{R195A}, Lane 3 represents VfmH^{R279A}, and Lane 4 represents VfmH^{R344A} proteins, respectively (M.W. 54.94 kDa). (B) Histidine-tagged YcgR and histidine-tagged maltose binding protein (MBP) were detected by mouse anti-His monoclonal antibody and goat anti-mouse HRP conjugate secondary antibody. Lane 1 represents histidine-tagged YcgR (M.W. 29.2kDa) and Lane 2 represents histidine-tagged MBP (M.W. 45.5kDa) proteins respectively. (C) MBP-tagged CRP protein expression from pET21b vector analyzed by SDS-PAGE and Coomassie staining. Lane 1 represents an uninduced sample, and lanes 2, 3, and 4 include MBP-CRP expression over time (1h, 3h, and 5h post-induction with IPTG respectively). The specific protein bands are labeled with black arrows.

Figure S8. Pi standard curve.

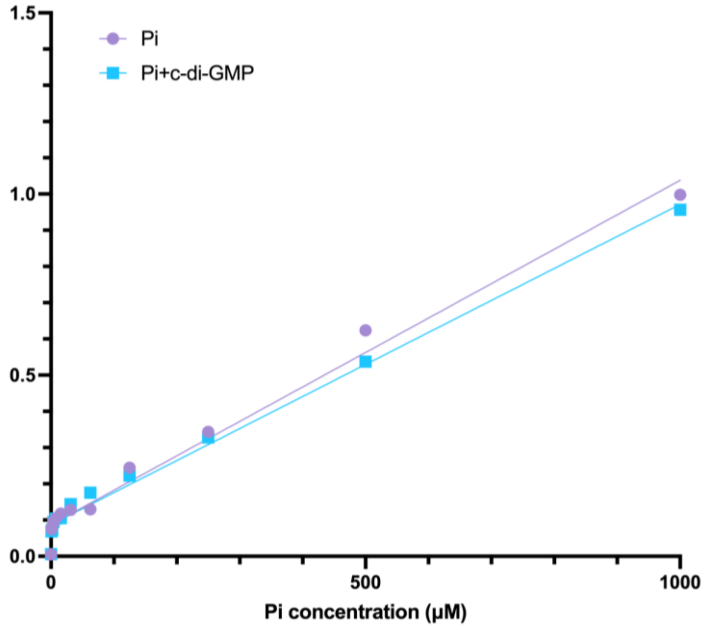

A standard curve of inorganic phosphate was constructed with different concentrations. The line in purple shows the absorbance at 620 nm with Malachite green added to different concentrations of Pi. The line in purple indicates Pi standard curve in presence of c-di-GMP (control).

References

1. Yi X, Yamazaki A, Biddle E, Zeng Q, Yang C-H. 2010. Genetic analysis of two phosphodiesterases reveals cyclic diguanylate regulation of virulence factors in *Dickeya dadantii*. Mol Microbiol 77:787–800.
2. Nasser W, Robert-Baudouy J, Reverchon S. 1997. Antagonistic effect of CRP end KdgR in the transcription control of the *Erwinia chrysanthemi* pectinolysis genes. Mol Microbiol 26:1071–1082.
3. Datsenko KA, Wanner BL. 2000. One-step inactivation of chromosomal genes in *Escherichia coli* K-12 using PCR products. Proceedings of the National Academy of Sciences 97:6640–6645.
4. Metcalf WW, Jiang W, Daniels LL, Kim SK, Haldimann A, Wanner BL. 1996. Conditionally replicative and conjugative plasmids carrying *lacZα* for cloning, mutagenesis, and allele replacement in bacteria. Plasmid 35:1–13.
5. Yuan X, Khokhani D, Wu X, Yang F, Biener G, Koestler BJ, Raicu V, He C, Waters CM, Sundin GW, Tian F, Yang CH. 2015. Cross-talk between a regulatory small RNA, cyclic-di-GMP signalling and flagellar regulator FlhDC for virulence and bacterial behaviours. Environ Microbiol 17:4745–4763.
6. Lerner CG, Inouye M. 1990. Low copy number plasmids for regulated low-level expression of cloned genes in *Escherichia coli* with blue/white insert screening capability. Nucleic Acids Res 18:4631.
7. Miller WG, Leveau JHJ, Lindow SE. 2000. Improved *gfp* and *inaZ* Broad-Host-Range Promoter-Probe Vectors. Molecular Plant-Microbe Interactions 13:1243–1250.
8. Peng Q, Yang S, Charkowski AO, Yap M-N, Steeber DA, Keen NT, Yang C-H. 2006. Population Behavior Analysis of *dspE* and *pelD* Regulation in *Erwinia chrysanthemi* 3937. Molecular Plant-Microbe Interactions 19:451–457.
9. Banerjee B, Zeng Q, Yu M, Hsueh BY, Waters CM, Yang C. 2022. Quorum-sensing master regulator VfmE is a c-di-GMP effector that controls pectate lyase production in the phytopathogen *Dickeya dadantii*, Microbiology Spectrum 10:e01805-21.

10. Matsuyama BY, Krasteva P V., Baraquet C, Harwood CS, Sondermann H, Navarro MVAS. 2016. Mechanistic insights into c-di-GMP-dependent control of the biofilm regulator FleQ from *Pseudomonas aeruginosa*. Proceedings of the National Academy of Sciences 113:E209–E218.
